# Fecal microbiota transplantation as a therapy for treating ulcerative colitis: an overview of systematic reviews

**DOI:** 10.1186/s12866-023-03107-1

**Published:** 2023-11-29

**Authors:** Haixia Liu, Jing Li, Jiaxin Yuan, Jinke Huang, Youqi Xu

**Affiliations:** 1 The Second Affiliated Hospital of Nanjing University of Chinese Medicine, Nanjing, China; 2 Guang’an Hospital of Traditional Chinese Medicine, Guang’an, China; 3 Tianjin University of Traditional Chinese Medicine, Tianjin, 301617 China; 4 Xiyuan Hospital of China Academy of Chinese Medical Sciences, Beijing, China

**Keywords:** Fecal microbiota transplantation, Ulcerative colitis, Evidence, Remission

## Abstract

**Aim:**

The current overview on published systematic reviews (SRs) and meta-analysis (MAs) aimed to systematically gather, evaluate, and synthesize solid evidence for using fecal microbiota transplantation (FMT) to treat ulcerative colitis (UC).

**Methods:**

Relevant articles published before January 2023 were collected from Web of Science, Embase, PubMed, and Cochrane Library. Two authors used Assessment of Multiple Systematic Reviews 2 (AMSTAR-2) tool, PRISMA checklists, and Grading of Recommendations, Assessment, Development, and Evaluation (GRADE) system were applied by two authors to independently evaluate the methodological quality, reporting quality, and evidence quality, respectively. Re-meta-analysis on the primary RCTs was conducted after excluding overlapping randomized controlled trials (RCTs).

**Results:**

Six SRs/MAs involving 12 primary RCTs and 544 participants were included. According to the AMSTAR-2 tool and PRISMA checklist, methodological quality and reporting quality of the included studies was overall satisfactory. The evidence quality of a great majority of outcomes was rated as moderate to high according to the GRADE system. Compared to placebo, the re-meta-analysis found a great advantage of use FMT in inducing combined clinical and endoscopic remission (OR 3.83 [2.31, 6.34]), clinical remission (3.31 [2.09, 5.25]), endoscopic remission (OR 3.75 [2.20, 6.39]), clinical response (OR 2.56 [1.64, 4.00]), and endoscopic response (OR 2.18 [1.12, 4.26]). Pooled data showed no significant difference in serious adverse events between patients receiving FMT and those receiving placebo (OR 1.53 [0.74, 3.19]). Evidence quality of the outcomes derived from re-meta-analysis was significantly higher after overcoming the limitations of previous SRs/MAs.

**Conclusion:**

In conclusion, moderate- to high-quality evidence supported a promising use of FMT to safely induce remission in UC. However, further trials with larger sample size are still required to comprehensively analyze the delivery route, total dosage, frequency, and donor selection in FMT.

## Introduction

Ulcerative colitis (UC) is a chronic non-specific inflammatory bowel disease that primarily affects distal colonic mucosa and submucosa [1]. UC is characterized by remission and alternating active phases, with abdominal pain, anemia, and bloody diarrhea as the main clinical manifestations. UC is correlated with an increased risk of colectomy [2], and patients with recurrent or persistent UC are more likely to develop colitis-associated cancer (CAC) [3]. The induction and maintenance of long-term stable remission are currently the main objectives in UC treatment in order to lower the possibility of relapse and prevent further development of CAC [4]. Mounting evidence have indicated the relation between the colonic microbiome and the pathogenesis of UC [1], but a majority of current treatments for UC still focus on immune system and pro-inflammatory factors rather than luminal microbial environment [5, 6]. Fecal microbiota transplantation (FMT) has been widely accepted as a highly effective treatment for persistent or resistant *Clostridium difficile* infection [7–9]. This also encourages researchers to consider FMT as a potential treatment for other illnesses that might be influenced by microbiota [10]. Given the critical role of the microbiome in UC and the fact that colonic ecosystem could be altered by FMT, there is growing interest in treating UC with FMT [11, 12].

Evidence derived from systematic reviews (SRs) and meta-analyses (MAs) are typically believed to be able to offer a solid foundation for clinical decision-making, but this is not always reliable because clinical decision-making process could be misled by poor-quality evidence [13]. Hence, it is necessary to systematically compile, assess, and synthesize evidence from numerous SRs/MAs on the same topic [14]. Compared to traditional SRs/MAs, an overview that minimizes duplication of information and presents findings from SRs/MAs in a uniform format could serve as a "friendly front end" for decision makers, healthcare professionals, and patients with UC [15]. In addition, an overview as such often focuses on the methodological aspects of SRs/MAs and can therefore guide future high-quality SRs/MAs by identifying potential risks of bias that could downgrade the quality of evidence [15]. A rising number of SRs/MAs have examined the efficacy and safety of using FMT in treating UC. Therefore, to systematically compile, assess and analyze evidence from multiple SRs/MAs on this particular topic, we carried out a comprehensive evaluation on the methodological quality, reporting quality and evidence quality of related SRs/MAs.

## Methods

### Registration and protocol

The methodology of this study was performed following the Cochrane Handbook [16]. The protocol was registered in the PROSPERO database. This overview was reported in accordance with the PRIOR statement [15].

### Inclusion and exclusion criteria

Studies adhering to the following criteria were included: (1) Randomized clinical trials (RCTs) that examined the potential of FMT in treating UC were enrolled by SRs/MAs; (2) Participants met internationally recognized criteria for the diagnosis of UC regardless of gender, age, ethnicity, or duration of diseases [17]; (3) FMT was an intervention of interest for UC treatment, with a control group consisting of placebo or conventional medication; (4) Combined clinical and endoscopic remission, clinical remission, clinical response, endoscopic remission, endoscopic response, and serious adverse events were considered as outcomes.

The exclusion criteria were as follows: (1) Reviews including non-RCTs; (2) Reviews that included both UC patients and Crohn’s disease (CD) patients; (3) Reviews that were not efficacy evaluations; (3) Publications (e.g. conference abstracts, letters, and comments) without complete data.

### Searching methods for identifying eligible reviews

Until January 14, 2023, papers published in Web of Science, PubMed, Embase, and Cochrane Library were comprehensively searched. In order to identify eligible studies, references to systematic reviews on the related subject were also reviewed. Specific searching strategy was adjusted in different databases. Table 1 shows the searching strategy on these databases.

**Table 1:** Search strategy for PubMed

Query	Search term
#1	Ulcerative colitis [Mesh]
#2	Ulcerative colitis [Title/Abstract] OR Colitis [Title/Abstract] OR UC [Title/Abstract] OR Inflammatory bowel disease [Title/Abstract] OR IBD [Title/Abstract] OR Ulcer colonitis [Title/Abstract] OR Idiopathic proctocolitis [Title/Abstract]
#3	#1 OR #2
#4	Fecal microbiota transplant [Mesh]
#5	Fecal microbiota transplant [Title/Abstract] OR Faecal microbiota transplant [Title/Abstract] OR Stool transplant [Title/Abstract] OR FMT [Title/Abstract] OR Fecal transfusion [Title/Abstract] OR Fecal bacteriotherapy [Title/Abstract]
#6	#4 OR #5
#7	Meta-Analysis as Topic [Mesh]
#8	Meta-analysis [Title/Abstract] OR Systematic review [Title/Abstract] OR Meta-analyses [Title/Abstract] OR Meta analysis [Title/Abstract] OR Metaanalysis [Title/Abstract]
#9	#7 OR #8
#10	#3 AND #6 AND #9

### Evaluation of eligible papers and data extraction

Endnote X9 was used to import the retrieved papers and delete duplicates. The titles and abstracts of these papers were independently read by two authors to select eligible papers under inclusion criteria. For the final inclusion, the papers were read in full text.

Data extraction was independently performed by two authors using predesigned forms. For the included SRs/MAs, characteristics of reviews (country, publication year, first author) and characteristics of design (interventions, comparisons, quality assessment tool) were extracted. For the enrolled RCTs, methodological characteristics, reporting characteristics, and findings (outcomes, conclusions) were extracted. For the primary RCTs of the included SRs/MAs, the following data were collected: first author, publication year, country, sample size, methodological characteristics, severity of the disease, interventions, comparisons, concomitant treatment, duration to follow-up, and treatment outcomes of each patient.

### Quality assessment

The Assessment of Multiple Systematic Reviews 2 (AMSTAR2) tool [18], Preferred Reporting Items for Systematic Reviews and Meta-Analyses (PRISMA) checklists [19], and Grading of Recommendations, Assessment, Development, and Evaluation (GRADE) system [20] were applied by two authors to independently evaluate methodological quality, reporting quality, and evidence quality, respectively. There are 16 items in AMSTAR-2, and 7 of them are critical items (2, 4, 7, 9, 11, 13 and 15). The PRISMA consists of 27 items, each of which is rated as “no” (not reported), “yes” (fully reported), or “partially yes” (partially reported). GRADE system was applied to assess the evidence quality for the outcomes from SRs/MAs in terms of limitations, imprecision, indirectness, inconsistencies, and publication bias.

### Data synthesis

Summary statistics from the included reviews were analyzed. It was difficult to avoid overlapping trials because the reviews included in this overview all focused on the same topic. Considering that there might be overlapping trials and participants, we analyzed the data of the primary RCTs of the included SRs/Mas and conducted a re-analysis using RevMan 5.4. Pooled effect was presented as an odds ratio (OR) with 95% confidence intervals (CIs) for dichotomous variables. The presence of statistical heterogeneity was assessed by the Cochran’s Q test (χ^2^) and reported as *I*^*2*^ [21]. Fixed-effects model was applied in meta-analysis when the *I*^*2*^ < 50%, otherwise a random-effects model was used [22, 23]. Subgroup and sensitivity analyses were further performed to investigate source of heterogeneity when the *I*^*2*^ was higher than 50%. When possible and appropriate, number of stool donors, protocol of FMT (pre-FMT treatment, mode, frequency, route of FMT delivery), and concomitant use of topical rectal therapy or biologics were included in planned subgroup analyses. When more than 10 studies were pooled for a given outcome, a funnel plot was used to explore publication bias [24, 25]. Furthermore, GRADE system was employed to assess evidence quality for the outcomes obtained from data synthesized from the primary RCTs.

## Results

### Results on the publication selection

**Fig. 1:**
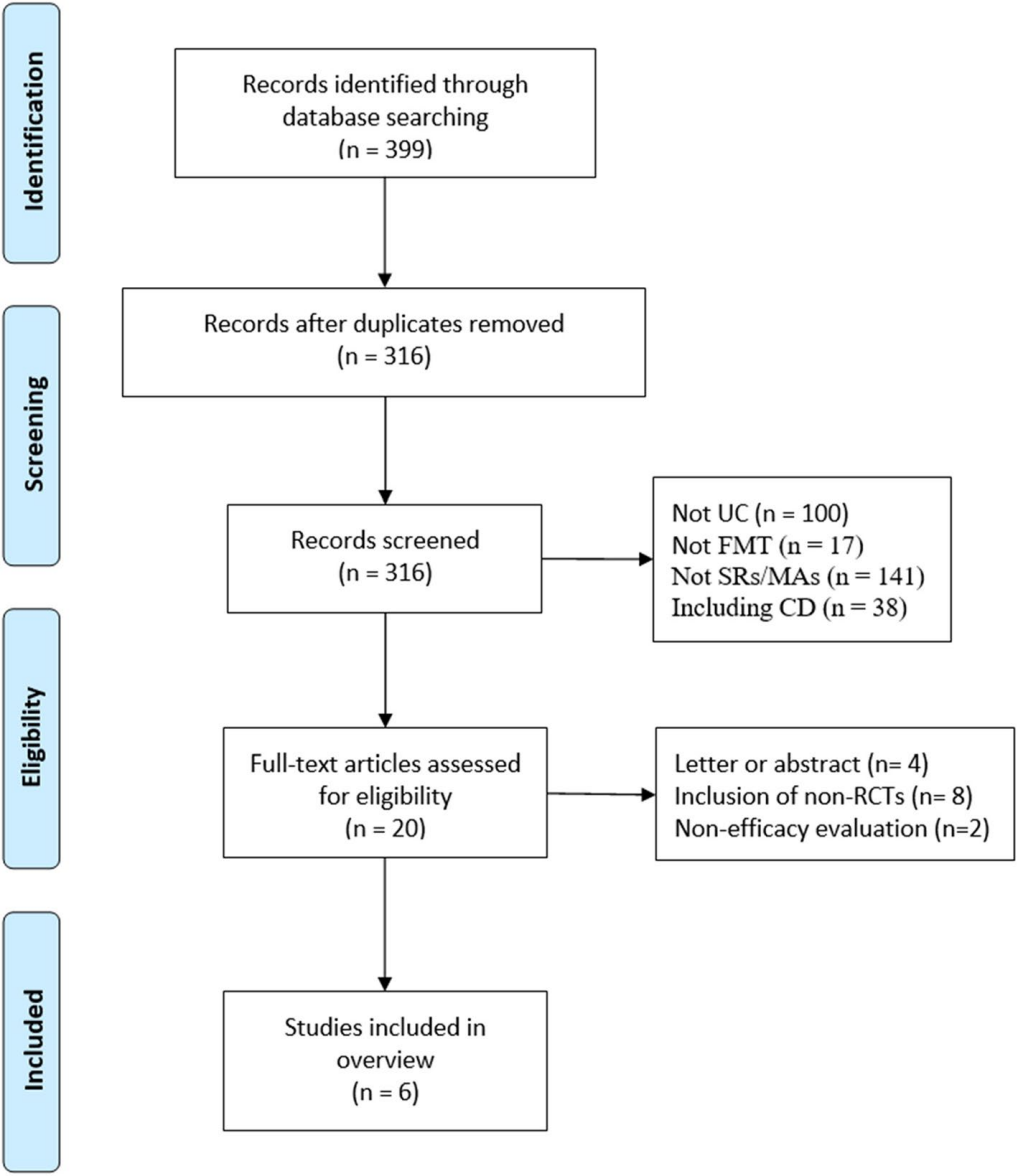
Flow-chart of study selection

In total, 399 publications were filtered, 279 of which were excluded after reviewing their abstracts and titles. Full text of the remaining publications was carefully read, and 13 of which were further excluded. Finally, 6 studies were considered to meet the inclusion criteria [26–31]. A flow chart of publication selection is shown in Fig. 1.

### Characteristics of the included studies

**Fig. 2:**
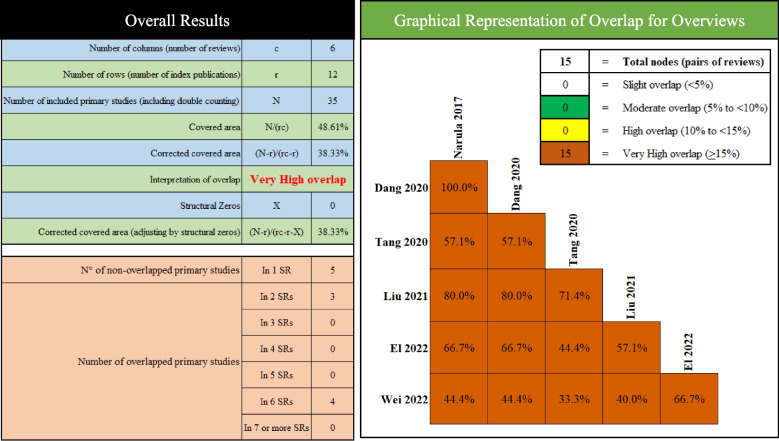
Overlap of trails included in reviews

Table 2 provides an overview on the characteristics of the included SRs/MAs. Studies included in this overview were published between 2017 and 2022. In all SRs/MAs, the searching was limited to RCT designs. Quality assessment was based on the Cochrane risk-of-bias tool (random sequence generation; allocation concealment; blinding of participants, personnel, and outcome assessors; incomplete outcome data; selective reporting; sponsorship; and other potential sources of bias). A total of 35 RCTs involving 2053 participants were included in these SRs/MAs. Notably, the CCA value was calculated to be 38.33%, indicating a noticeably high overlapping of RCTs included in these SRs/MAs (Fig. 2).

**Table 2:** Characteristics of the included reviews

Study	Country	Trials (subjects)	Experimental Intervention	Control Intervention	Quality assessment	Meta-analyses	Results summary
Narula, 2017 [26]	Canada	4 (277)	FMT	Placebo	Cochrane risk-of-bias tool	Yes	Among RCTs, short-term use of FMT shows promise as a treatment to induce remission in active UC based on the efficacy and safety observed
Dan, 2020 [27]	China	4 (277)	FMT	Placebo	Cochrane risk-of-bias tool	Yes	FMT achieved good results in clinical remission and clinical response in active ulcerative colitis, and there was no increased risk of adverse reactions
Tang, 2020 [28]	China	7 (431)	FMT	Placebo	Cochrane risk-of-bias tool	Yes	The results showed that FMT had better efficacy than placebo, frozen faeces from multiple donors delivered via the lower gastrointestinal tract had a better curative effect than placebo; the difference in efficacy between mixed faeces from a single donor transplanted through the upper gastrointestinaltract and placebo was not significant
Liu, 2021 [29]	China	5 (292)	FMT	Placebo,	Cochrane risk-of-bias tool	Yes	In conclusion, this review showed advantage of FMT over controls in clinical remission, endoscopic remission, and combined them together in patients with active UC. In addition, the lower gastrointestinal route of delivery, pooled donor stool, and higher frequency of administration may be more effective
El, 2022 [30]	USA	6 (324)	FMT	Placebo	Cochrane risk-of-bias tool	Yes	FMT is a safe and effective therapeutic modality for the induction of endoscopic and clinical remission of patients with UC compared with placebo and with a good safety profile
Wei, 2022 [31]	China	9 (452)	FMT	Placebo, UC exclusion diet, 5-ASA enema	Cochrane risk-of-bias tool	Yes	This meta-analysis of RCTs showed that FMT had significant advantages in terms of clinical and endoscopic remission in patients with mild to moderate active UC

After removing overlapping RCTs (23 RCTs with 1509 participants), there  remained 12 primary RCTs [32–43] with 544 participants. Table 3 provides an overview of characteristics of the primary RCTs of the included SRs/MAs. All the 12 primary RCTs used FMT as experimental intervention, but 10 of them set placebo as control intervention and two RCTs used 5-aminosalicylate enema (1 RCT with 43 participants) and dietary treatment with an UC exclusion diet (1 RCT with 34 participants) as control intervention.

**Table 3:** Characteristics of the primary RCTs of included reviews

Study	Country	No. Sample size (n1/n2)	Severity	Intervention of FMT	Intervention of control	Concomitant treatment	Duration to follow-up	Combined clinical and endoscopic remission n/response (n1/n2)	Clinical remission/response (n1/n2)	Endoscopic remission/response (n1/n2)	Serious adverse events (n1/n2)
Rossen, 2015 [32]	Netherlands	23/25	SCCAI score 4–11	Fresh single-donor FMT; administered through nasoduodenal tube at time 0 then at week 3	Autologous stool administered through nasoduodenal tube	Stable maintenance medication (5-ASA, thiopurines), prednisolone #10 mg/d	Week 12	7/5	18/21	10/11	2/2
Moayyedi, 2015 [33]	Canada	38/37	Mayo score ≥ 4	Fresh and frozen single-donor FMT; administered through weekly enema	Water retention enema	Stable maintenance medication (5-ASA, thiopurines, methotrexate, anti-TNF, steroids)	Week 7	15/9	24/11	9/3	3/2
Costello, 2019 [34]	Australia	38/35	Mayo score 3–10	Frozen pooled-donors FMT; administered by means of colonoscopy at time 0, then 2 enemas at day 7	Aerobically prepared, autologous stool; administered by means of colonoscopy at time 0, then 2 enemas at day 7	Stable maintenance medication (5-ASA, thiopurines, methotrexate, anti-TNF, vedolizumab), prednisolone #20 mg/d with mandatory wean	Week 8	12/3	39/13	21/6	3/2
Paramsothy, 2017 [35]	Australia	41/40	Mayo score 4–10	Frozen pooled-donors FMT; administered by means of colonoscopy at time 0, then 5 enemas per week for 8 weeks	Saline + odorant + food colouring in enema	Stable maintenance medication (5-ASA, thiopurines, methotrexate), prednisolone #20 mg/d with mandatory wean	Week 8	11/3	40/17	18/7	2/1
Crothers, 2018 [36]	USA	7/8	Mayo score 4–10	Frozen and fresh pooled-donors FMT; administered by means of colonoscopy at time 0, followed by daily FMTc (0.375 g stool)	Sham FMT and daily placebo capsules	N/A	Week 18	3/0	NR	6/0	NR
Mahajan, 2018 [37]	India	22/21	Mayo score 4–10	Administered by means of colonoscopy	Placebo	N/A	Week 48	18/8	19/14	NR	NR
Sood, 2019 [38]	India	31/30	Mayo score 4–10	Frozen single-donor FMT; administered by means of colonoscopy at weeks 0, 8, 16, 24, 32, 40 and 48	Placebo, administered by means of colonoscopy at weeks 0, 8, 16, 24, 32, 40 and 48	Stable maintenance medication (5-ASAs, corticosteroids, thiopurines)	Week 48	NR	27/20	18/8	0/1
Crothers, 2021 [39]	USA	6/6	Mayo score 4–10	Frozen single-donor FMT; administered by means of colonoscopy at weeks 0, followed by a dose of 1 daily 550μL FMT capsule, for 12 weeks	Sham colonoscopic infusion and sham capsules	Stable maintenance medication (anti-TNFα, immunomodulators, 5-ASA, methotrexate)	Week 12	2/0	5/3	NR	1/1
Březina, 2021 [40]	Czech Republic	21/22	Mayo score 4–10	Frozen single-donor FMT; administered 10 FMT infusions, five in the first week and once weekly in the following 5 weeks	A standard-of-care regimen that included 4 g mesalamine enemas daily for 2 weeks and then every other day until the end of week 6	Stable maintenance medication (5-ASAs, prednisone, thiopurines)	Week 12	NR	27/20	3/3	22/21
Pai, 2021 [41]	Canada	12/12	N/A	Frozen pooled-donors FMT; retention enema	Placebo	Stable maintenance medication (anti-TNFα, immunomodulator)	Week 30	NR	5/4	NR	5/1
Sarbagili, 2022 [42]	Israel	19/15	SCCAI score 5–11	Frozen single-donor FMT; administered by means of colonoscopy on day 1, then revived rectal enemas from the same donor on days 2 and 14	With an ulcerative colitis exclusion diet for 12 weeks	Stable maintenance medication (5-ASA, steroids, biologics, immunomodulators)	Week 8	NR	4/6	3/4	0/0
Haifer, 2022 [43]	Australia	15/20	Mayo score 4–10	Frozen single-donor FMT; oral lyophilised FMT for 8 weeks	Oral placebo capsules for 8 weeks	Stable maintenance medication (5-ASA, thiopurines, methotrexate, prednisolone, biologics)	Week 8	8/3	22/14	15/11	2/1

*n1* faecal microbiota transplantation group (FMT group), *n2* control group, *FMT* Fecal microbiota transplantation, *SCCAI* Simple clinical colitis activity index, *NR* No record, *5-ASA* 5-aminosalicylates, *TNF* Tumour necrosis factor, *MTX* Methotrexate

### Results of the methodological quality assessment

The AMSTAR-2 assessment of the methodological quality is shown in Table 4. Two included studies met all the critical items of AMSTAR-2 and were therefore rated at as having a high methodological quality. The remaining 4 studies were rated as having a moderate methodological quality because they did not provide a list of excluded trials. Overall, the methodological quality of the included studies was satisfactory.

**Table 4:** Methodological quality of the included reviews

Author, Year	AMSTAR-2																Quality
	**Q1**	**Q2**	**Q3**	**Q4**	**Q5**	**Q6**	**Q7**	**Q8**	**Q9**	**Q10**	**Q11**	**Q12**	**Q13**	**Q14**	**Q15**	**Q16**	
Narula, 2017 [26]	Y	Y	Y	Y	Y	Y	Y	Y	Y	Y	Y	Y	Y	Y	Y	Y	High
Dang, 2020 [27]	Y	Y	Y	Y	Y	Y	Y	Y	Y	Y	Y	Y	Y	Y	Y	Y	High
Tang, 2020 [28]	Y	Y	Y	Y	Y	Y	PY	Y	Y	Y	Y	Y	Y	Y	Y	Y	Moderate
Liu, 2021 [29]	Y	Y	Y	Y	Y	Y	PY	Y	Y	Y	Y	Y	Y	Y	Y	Y	Moderate
El, 2022 [30]	Y	Y	Y	Y	Y	Y	PY	Y	Y	Y	Y	Y	Y	Y	Y	Y	Moderate
Wei, 2022 [31]	Y	Y	Y	Y	Y	Y	PY	Y	Y	Y	Y	Y	Y	Y	Y	Y	Moderate

*Y* Yes, *PY* Partial Yes, *N* No

### Results of the reporting quality assessment

The reporting quality assessed by PRISMA is presented in Table 5. Although no studies reported all 42 the items, all of them reported over 88% of the PRISMA checklists. Overall, the reporting quality of the included studies was satisfactory. However, poor reporting quality was common in additional analyses such as sensitivity (items 13f and 20d), summary of the main findings including certainty of the evidence (items 15 and 22), and lack of registration (item 24a).

**Table 5:** Reporting quality of the included reviews

**Section/ topic**	**Items**	**Narula, 2017 **[26]	**Dang, 2020 **[27]	**Tang, 2020 **[28]	**Liu, 2021 **[29]	**El, 2022 **[30]	**Wei, 2022 **[31]	**Compliance (%)**
Title	1	Y	Y	Y	Y	Y	Y	100%
Abstract	2	Y	Y	Y	Y	Y	Y	100%
Introduction	3	Y	Y	Y	Y	Y	Y	100%
	4	Y	Y	Y	Y	Y	Y	100%
Methods	5	Y	Y	Y	Y	Y	Y	100%
	6	Y	Y	Y	Y	Y	Y	100%
	7	Y	Y	Y	Y	Y	Y	100%
	8	Y	Y	Y	Y	Y	Y	100%
	9	Y	Y	Y	Y	Y	Y	100%
	10a	Y	Y	Y	Y	Y	Y	100%
	10b	Y	Y	Y	Y	Y	Y	100%
	11	Y	Y	Y	Y	Y	Y	100%
	12	Y	Y	Y	Y	Y	Y	100%
	13a	Y	Y	Y	Y	Y	Y	100%
	13b	Y	Y	Y	Y	Y	Y	100%
	13c	Y	Y	Y	Y	Y	Y	100%
	13d	Y	Y	Y	Y	Y	Y	100%
	13e	Y	Y	Y	Y	Y	Y	100%
	13f	N	N	N	N	Y	N	16.7%
	14	Y	Y	Y	Y	Y	Y	100%
	15	Y	N	N	N	N	N	16.7%
Results	16a	Y	Y	Y	Y	Y	Y	100%
	16b	Y	Y	Y	Y	Y	Y	100%
	17	Y	Y	Y	Y	Y	Y	100%
	18	Y	Y	Y	Y	Y	Y	100%
	19	Y	Y	Y	Y	Y	Y	100%
	20a	Y	Y	Y	Y	Y	Y	100%
	20b	Y	Y	Y	Y	Y	Y	100%
	20c	Y	Y	Y	Y	Y	Y	100%
	20d	N	N	N	N	Y	N	16.7%
	21	Y	Y	Y	Y	Y	Y	100%
	22	Y	N	N	N	N	N	16.7%
Discussion	23a	Y	Y	Y	Y	Y	Y	100%
	23b	Y	Y	Y	Y	Y	Y	100%
	23c	Y	Y	Y	Y	Y	Y	100%
	23d	Y	Y	Y	Y	Y	Y	100%
Other information	24a	N	N	N	N	N	N	0.00%
	24b	Y	Y	Y	Y	Y	Y	100%
	24c	Y	Y	Y	Y	Y	Y	100%
	25	Y	Y	Y	Y	Y	Y	100%
	26	Y	Y	Y	Y	Y	Y	100%
	27	Y	Y	Y	Y	Y	Y	100%

*Y* Yes, *N* No

### Results of the evidence quality assessment

Evidence quality of outcomes from the included studies was evaluated by the GRADE system (Table 6). Among the 22 outcomes, 8 showed high-quality confidence (36.4%), 12 showed moderate-quality confidence (54.5%), and 2 two showed low-quality confidence (9.1%). Small sample size in the existing studies were the most common factor for downgrading the evidence quality.

**Table 6:** Evidence quality of the included reviews

**Review**	**Outcomes**	**№ of trials**	**Design**	**Certainty assessment**					**№ of patients**		**Relative effect (95% CI)**	**Quality**
				**Limitations**	**Inconsistency**	**Indirectness**	**Imprecision**	**Publication bias**	**Experimental**	**Control**		
Narula, 2017 [26]	CER	4	Rct	No	No	No	Serious^c^	No	140	137	RR 2.97 [1.66, 5.33]	⨁⨁⨁⨁◯ Moderate
	Clinical remission	4	Rct	No	No	No	Serious^c^	No	140	137	RR 1.87 [1.29, 2.70]	⨁⨁⨁⨁◯ Moderate
	Endoscopic remission	4	Rct	No	Serious^b^	No	Serious^c^	No	140	137	RR 3.26 [1.90, 5.59]	⨁⨁⨁◯◯ Low
	ADs	4	Rct	No	No	No	Serious^c^	No	140	137	RR 1.40 [0.55, 3.58]	⨁⨁⨁⨁◯ Moderate
Dang, 2020 [27]	Clinical remission	4	Rct	No	No	No	Serious^c^	No	140	137	OR 3.47 [1.93, 6.25]	⨁⨁⨁⨁◯ Moderate
	Clinical response	4	Rct	No	Serious^b^	No	Serious^c^	No	140	137	OR 2.48 [1.18, 5.21]	⨁⨁⨁◯◯ Low
	ADs	4	Rct	No	No	No	Serious^c^	No	140	137	OR 1.29 [0.46, 3.57]	⨁⨁⨁⨁◯ Moderate
Tang, 2020 [28]	Clinical remission	7	Rct	No	No	No	No	No	217	214	OR 2.29 [1.48, 3.53]	⨁⨁⨁⨁⨁ High
	ADs	6	Rct	No	No	No	No	No	210	206	RR 1.37 [0.63, 2.96]	⨁⨁⨁⨁⨁ High
Liu, 2021 [29]	CER	5	Rct	No	No	No	Serious^c^	No	147	145	RR 3.14 [1.78, 5.55]	⨁⨁⨁⨁◯ Moderate
	Clinical remission	5	Rct	No	No	No	Serious^c^	No	147	145	RR 1.85 [1.28, 2.67]	⨁⨁⨁⨁◯ Moderate
	Endoscopic remission	5	Rct	No	No	No	Serious^c^	No	147	145	RR 2.26 [1.28, 5.53]	⨁⨁⨁⨁◯ Moderate
	ADs	5	Rct	No	No	No	Serious^c^	No	147	145	RR 0.98 [0.93, 1.03]	⨁⨁⨁⨁◯ Moderate
El, 2022 [30]	CER	6	Rct	No	No	No	No	No	161	163	OR 4.11 [2.19,7.72]	⨁⨁⨁⨁⨁ High
	Clinical remission	4	Rct	No	No	No	Serious^c^	No	117	120	OR 3.06 [1.35, 6.89]	⨁⨁⨁⨁◯ Moderate
	Clinical response	6	Rct	No	No	No	No	No	161	163	OR 2.60 [1.54, 4.40]	⨁⨁⨁⨁⨁ High
	Endoscopic remission	4	Rct	No	No	No	Serious^c^	No	132	132	OR 3.78 [1.59, 8.97]	⨁⨁⨁⨁◯ Moderate
	Endoscopic response	3	Rct	No	No	No	Serious^c^	No	79	95	OR 2.17 [1.05, 4.50]	⨁⨁⨁⨁◯ Moderate
	ADs	6	Rct	No	No	No	No	No	161	163	OR 1.38 [0.58, 3.30]	⨁⨁⨁⨁⨁ High
Wei, 2022 [31]	Clinical remission	9	Rct	No	No	No	No	No	213	212	RR 1.84 [1.37, 2.47]	⨁⨁⨁⨁⨁ High
	Endoscopic remission	7	Rct	No	No	No	No	No	195	194	RR 1.94 [1.14, 3.31]	⨁⨁⨁⨁⨁ High
	ADs	9	Rct	No	No	No	No	No	213	212	RR 2.05 [1.03, 4.09]	⨁⨁⨁⨁⨁ High

*CER* Combined clinical and endoscopic remission, *ADs* Severe adverse events

^a^The experimental design had a large bias in random, distributive findings or was blind

^b^The confidence interval overlaps less, the heterogeneity test *P* was very small, and the *I*^*2*^ was larger

^c^The Confidence interval was not narrow enough, or the simple size is too small

^d^Funnel graph asymmetry, or fewer studies were included and there may have been greater publication bias

### Efficacy and safety of interventions

#### Reported efficacy and safety of FMT from the included reviews

Combined clinical and endoscopic remission of UC patients receiving FMT or placebo was assessed in 3 reviews, and their results showed a significantly greater benefit for patients receiving FMT than placebo (RR 2.97 [1.66, 5.33]; RR 3.14 [1.78, 5.55]; OR 4.11 [2.19,7.72]). FMT was reported to have a significant effect on endoscopic remission compared to the controls in 4 reviews (RR 3.26 [1.90, 5.59]; RR 2.26 [1.28, 5.53]; OR 3.78 [1.59, 8.97]; RR 1.94 [1.14, 3.31]). FMT also achieved significant clinical remission compared to the controls in all the reviews (RR 1.87 [1.29, 2.70]; OR 3.47 [1.93, 6.25]; OR 2.29 [1.48, 3.53]; RR 1.85 [1.28, 2.67]; OR 3.06 [1.35, 6.89]; RR 1.84 [1.37, 2.47]). Furthermore, patients treated by FMT showed more endoscopic responses than those receiving control therapy in 1 review (OR 2.17 [1.05, 4.50]). Additionally, clinical response of UC patients treated by FMT or placebo was assessed in 2 reviews, which manifested a significantly greater benefit for patients receiving FMT than placebo (OR 2.48 [1.18, 5.21]; OR 2.17 [1.05, 4.50]).

Rate of serious adverse events showed no significant statistical significance in between the FMT and control groups in 5 reviews (RR 1.40 [0.55, 3.58]; OR 1.29 [0.46, 3.57]; RR 1.37 [0.63, 2.96]; RR 0.98 [0.93, 1.03]; OR 1.38 [0.58, 3.30]). However, 1 study found that 10.3% of the participants in the FMT group experienced serious adverse events in comparison to 5.19% of the participants in the control group (RR 2.05 [1.03, 4.09]).

#### Reported efficacy and safety of FMT from re-analysis of the primary trials

**Fig. 3:**
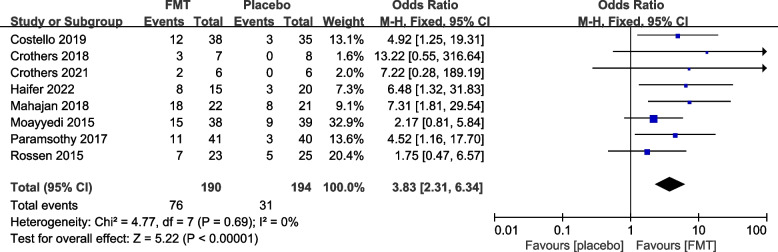
Forest plot comparing the rate of combined clinical and endoscopic remission in patients with UC receiving FMT vs placebo

**Fig. 4:**
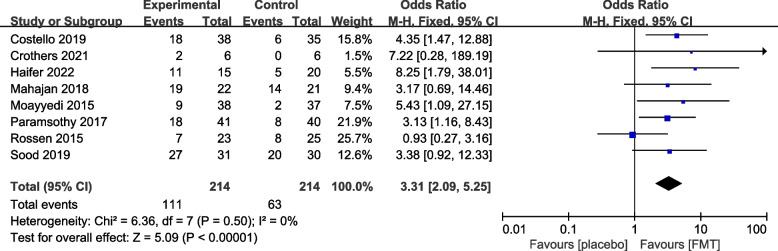
Forest plot comparing the rate of clinical remission in patients with UC receiving FMT vs placebo

**Fig. 5:**
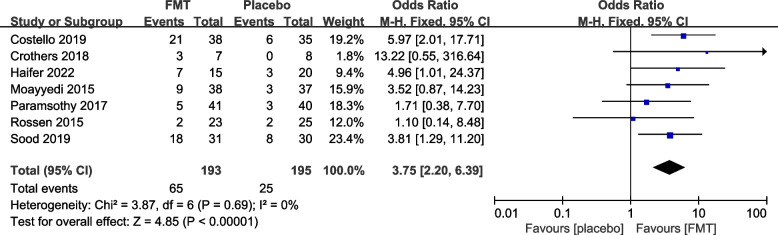
Forest plot comparing the rate of endoscopic remission in patients with UC receiving FMT vs placebo

**Fig. 6:**
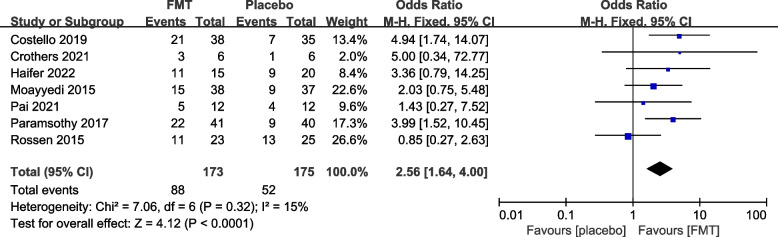
Forest plot comparing the rate of clinical response in patients with UC receiving FMT vs placebo

**Fig. 7:**
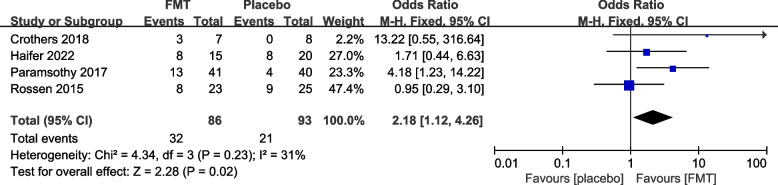
Forest plot comparing the rate of endoscopic response in patients with UC receiving FMT vs placebo. CI, confidence interval; M-H, Mantel–Haenszel

**Fig. 8:**
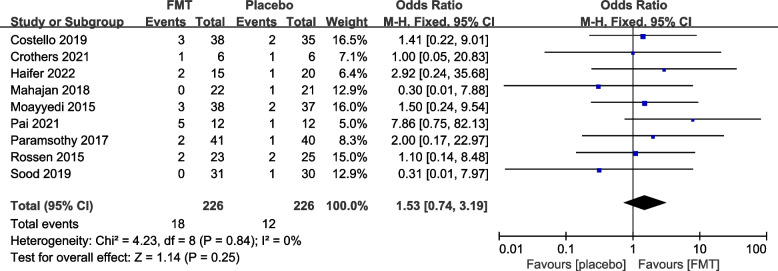
Forest plot comparing the rate of serious adverse events in patients with UC receiving FMT vs placebo

The 12 primary RCTs with 544 participants were used to perform an additional meta-analysis. Combined clinical and endoscopic remission was reported in 8 trials involving 384 patients, and the pooled results demonstrated a significantly greater benefit for patients receiving FMT than placebo (OR 3.83 [2.31, 6.34]) (Fig. 3). Clinical remission was reported in 8 RCTs with 428 patients, and the pooled results showed that significantly more patients in the FMT group (51.87%) had clinical remission (3.31 [2.09, 5.25]) than the control group (16.14%) (Fig. 4). Similarly, endoscopic remission was reported in 7 trials with 388 patients, and the pooled analysis revealed that 33.69% of patients in the FMT group achieved endoscopic remission in comparison to 12.82% in the placebo group (OR 3.75 [2.20, 6.39]) (Fig. 5). Clinical response was reported in 7 RCTs with 348 patients, and the pooled results demonstrated a significantly greater benefit for patients receiving FMT than placebo (OR 2.56 [1.64, 4.00]) (Fig. 6). Furthermore, 4 trials with 179 patients evaluated endoscopic response, and the pooled analysis revealed that 37.21% of patients in the FMT group and 22.58% of patients in the control group had endoscopic response (OR 2.18 [1.12, 4.26]) (Fig. 7). Additionally, rate of serious adverse events was reported in both primary trials, and no statistical difference between the FMT and control groups was shown in the pooled results (OR 1.53 [0.74, 3.19]) (Fig. 8).

Furthermore, evidence quality from re-analysis of the outcomes were further evaluated using GRADE system (Table 7). In summary, all the outcomes reached positive conclusion, and the outcomes of combined clinical and endoscopic remission, clinical remission, clinical response, endoscopic remission, and serious adverse events showed a high-quality confidence. Endoscopic response showed a moderate-quality confidence due to a small sample size in the available trials.

**Table 7:** Evidence quality of re-analysis on FMT for UC with data from primary trials

**Outcomes**	**№ of trials**	**Certainty assessment**					**№ of patients**		**OR (95% CI)**	**Quality**
		**Limitations**	**Inconsistency**	**Indirectness**	**Imprecision**	**Publication bias**	**Experimental**	**Control**		
CER	8	No	No	No	No	No	190	194	3.83 [2.31, 6.34]	⨁⨁⨁⨁⨁ High
Clinical remission	8	No	No	No	No	No	214	214	3.31 [2.09, 5.25]	⨁⨁⨁⨁⨁ High
Endoscopic remission	7	No	No	No	No	No	193	195	3.75 [2.20, 6.39]	⨁⨁⨁⨁⨁ High
Clinical response	7	No	No	No	No	No	173	175	2.56 [1.64, 4.00]	⨁⨁⨁⨁⨁ High
Endoscopic response	4	No	No	No	Serious^c^	No	86	93	2.18 [1.12, 4.26]	⨁⨁⨁⨁◯ Moderate
ADs	9	No	No	No	No	No	226	226	1.53 [0.74, 3.19]	⨁⨁⨁⨁⨁ High

*CER* Combined clinical and endoscopic remission, *Ads* Severe adverse events

^a^The experimental design had a large bias in random, distributive findings or was blind

^b^The confidence interval overlaps less, the heterogeneity test *P* was very small, and the *I*^*2*^ was larger

^c^The Confidence interval was not narrow enough, or the simple size is too small

^d^Funnel graph asymmetry, or fewer studies were included and there may have been greater publication bias

## Discussion

The number of SRs/MAs investigating FMT as a treatment for UC has shown an increase in recent years [26–31]. These studies had varying degrees of overlaps in terms of inclusion of trials, interventions, comparisons, and outcomes, and their results were not always organized in a consistent manner. Therefore, a more comprehensive overview is required to improve the current understanding of the effectiveness and safety of using FMT for treating UC.

### Summary of main results

In this study, data from 35 RCTs with a total of 2053 participants were synthesized to provide evidence for the effectiveness and safety of using FMT to treat UC. Firstly, evidence from the included reviews indicated that FMT better improved the combined clinical and endoscopic remission, clinical response, endoscopic remission, endoscopic response, and clinical remission compared to the control group. However, results of SRs/MAs on serious adverse events related to the safety of FMT in UC were inconsistent. Secondly, the included SRs/MAs had less serious methodological flaws or reporting gaps, with an overall satisfactory quality evaluated by the AMSTAR-2 tool and PRISMA checklists. Thirdly, using GRADE system, we found that the overall evidence quality was unsatisfactory, which was mainly due to a small sample size in the previous studies that may lower the evidence confidence. Therefore, the conclusions reached by the included studies differed from the actual results. Fourthly, given the considerable overlap among these reviews and a small sample size, we performed an additional meta-analysis containing more primary trials than individual SRs/MAs (12 primary trials, 544 participants). The pooled analysis revealed that FMT better improved the combined clinical and endoscopic remission, endoscopic remission, clinical remission, endoscopic response, and clinical response than using placebo. Interestingly, after expanding the number of trials and sample size, the pooled analysis results showed that the conflicting outcomes in the incidence of serious adverse events were confirmed to be similar when using FMT or placebo. Additionally, the evidence quality on the outcomes derived from the additional meta-analysis was significantly higher after expanding the number of trials and sample size.

### Overall completeness and applicability of evidence

The current overview included the latest studies published before January 2023, most of which were published within the last three years. UC patients in all the reviews were included regardless of their age, gender, or severity of illness. Outcomes included in the reviews were also relatively comprehensive. All the primary outcomes and most secondary outcomes in each review were included. Importantly, all reviews were consistent in their conclusions regarding the effects of FMT in UC, and incidence of serious adverse events were confirmed by additional meta-analyses with more trials and expanded sample sizes.

Various FMT protocols placed restrictions on the overall completeness and applicability of the evidence. Although no statistically significant heterogeneity was found in the previous SR/MA and current additional meta-analyses, there were differences between the main study experiments in terms of delivery route, total dosage, frequency, and donor selection. Fecal quality of donors, quantity of infusions, and mode of administration all affect how FMT works [44]. To date, the protocol of FMT has not been standardized [44]. Therefore, more trials are needed to determine the optimal timing, total dosage, frequency, delivery route, and the most suitable donor for FMT.

### Implications for research

The GRADE system was applied to evaluate the certainty of evidence for using FMT to treat UC, where the majority of comparisons were rated as moderate or low, suggesting that additional studies may have a significant impact on the level of confidence in the estimate of effect or could even change estimate. As for sample size, the vast majority of the primary trials included were considered as having a small sample size, which was the main reason for downgrading the certainty of evidence of primary trials. Small trials could increase the risk of small-trail biases and relevant issues of publication bias, because small negative trials are less likely to reach full publication, which can result in overly positive results in comparisons [45, 46]. Thus, more future trials using a standard FMT protocol (such as best timing, total dosage, frequency, delivery route, and the best donor) with greater participant motivation and larger sample size are needed.

## Strengths and limitations

To the best of our knowledge, this was the first overview of SRs/Mas that investigated the efficacy of using FMT for UC treatment. By systematically collecting, evaluating, and synthesizing the evidence for FMT for UC, the findings of this study could facilitate evidence-based decision-making process [47]. However, assessing methodological quality, reporting quality, and evidence quality could be a subjective process, as a result, outcomes may differ depending on the decisions made by various researchers in evaluating each factor. Furthermore, reviews that included both UC patients and Crohn’s disease patients were excluded, which may lead to selection bias.

## Conclusion

In conclusion, moderate- to high-quality evidence supported a promising use of FMT to safely induce remission in UC. However, various FMT protocols placed restrictions to the overall completeness and applicability of the evidence because fecal quality of donors, quantity of infusions, and mode of administration could all affect FMT. Therefore, further trials with larger sample sizes are needed to analyze the delivery route, total dose, frequency and donor selection of FMT, so as to develop a mature standardized protocol for FMT in clinical application.

## Data Availability

The datasets used and analyzed during the current study were available from the corresponding author on reasonable request.
